# Characterization of LDD-2633 as a Novel RET Kinase Inhibitor with Anti-Tumor Effects in Thyroid Cancer

**DOI:** 10.3390/ph14010038

**Published:** 2021-01-06

**Authors:** Hyo Jeong Lee, Pyeonghwa Jeong, Yeongyu Moon, Jungil Choi, Jeong Doo Heo, Yong-Chul Kim, Sun-Young Han

**Affiliations:** 1 College of Pharmacy and Research Institute of Pharmaceutical Sciences, Gyeongsang National University, Jinju-si 52828, Korea; witch816@nate.com; 2 Biomedical Science and Engineering and School of Life Sciences, Gwangju Institute of Science & Technology, Gwangju 61005, Korea; vor.peace@gmail.com (P.J.); yongchul@gist.ac.kr (Y.-C.K.); 3 Gyeongnam Department of Environmental Toxicology and Chemistry, Korea Institute of Toxicology, Jinju-si 52834, Korea; ygmoon@kitox.re.kr (Y.M.); jungil.choi@kitox.re.kr (J.C.); jdher@kitox.re.kr (J.D.H.)

**Keywords:** RET, thyroid cancer, LDD-2633, kinase inhibitors

## Abstract

Rearranged during transfection (RET), a receptor tyrosine kinase, is activated by glial cell line-derived neurotrophic factor family ligands. Chromosomal rearrangement or point mutations in *RET* are observed in patients with papillary thyroid and medullary thyroid carcinomas. Oncogenic alteration of *RET* results in constitutive activation of RET activity. Therefore, inhibiting RET activity has become a target in thyroid cancer therapy. Here, the anti-tumor activity of a novel RET inhibitor was characterized in medullary thyroid carcinoma cells. The indirubin derivative LDD-2633 was tested for RET kinase inhibitory activity. In vitro, LDD-2633 showed potent inhibition of RET kinase activity, with an IC_50_ of 4.42 nM. The growth of TT thyroid carcinoma cells harboring an RET mutation was suppressed by LDD-2633 treatment via the proliferation suppression and the induction of apoptosis. The effects of LDD-2633 on the RET signaling pathway were examined; LDD-2633 inhibited the phosphorylation of the RET protein and the downstream molecules Shc and ERK1/2. Oral administration of 20 or 40 mg/kg of LDD-2633 induced dose-dependent suppression of TT cell xenograft tumor growth. The in vivo and in vitro experimental results supported the potential use of LDD-2633 as an anticancer drug for thyroid cancers.

## 1. Introduction

Thyroid cancers are categorized into three main histological types: differentiated, medullary, and anaplastic cancers [1]. Histologically differentiated papillary thyroid carcinoma (PTC) is the most common type of thyroid cancer and accounts for approximately 80% of all thyroid malignancies [2]. Medullary thyroid carcinoma (MTC) originates from calcitonin-producing cells and accounts for approximately 4% of all malignant thyroid tumors [3]. The main treatment option for thyroid carcinoma is surgery, and radioactive iodine therapy can be employed as adjuvant therapy for differentiated thyroid cancer. The 10-year survival rate of patients with differentiated thyroid carcinoma in the USA is high, that is, more than 90% [1]. However, the 10-year survival rate is less than 10% for patients with radioiodine-refractory differentiated thyroid cancer [4]. For unresectable, progressive, and metastatic MTC, the median 10-year survival rate is 40% [5]. Treatment of thyroid carcinoma remains challenging, and targeted therapy utilizing small-molecule inhibitors is a prospective treatment alternative. Oncogenic alteration of *RET* has been reported in both PTC and MTC; thus, the RET protein has become a promising target for thyroid carcinoma therapies [6].

The *RET* proto-oncogene encodes a transmembrane receptor tyrosine kinase that is involved in oncogenesis in various cancers [2]. In normal cells, RET kinase is activated by glial cell line-derived neurotrophic factor (GDNF) family ligands. These ligands do not directly bind to the RET; instead, their binding is mediated by a co-receptor: the GDNF family receptor-α. Upon the activation of the RET kinase, signals are mediated by transduction pathways, such as the mitogen-activated protein kinase (MAPK) and phosphoinositide 3-kinase pathways. Oncogenic alterations in RET proteins result from chromosomal rearrangement or mutations in the *RET* sequence. Chromosomal rearrangement results in the fusion of a protein in the RET kinase domain with a partner protein. In cancers, the commonly found fusion proteins are coiled-coil domains containing 6-RET, nuclear receptor coactivator 4-RET, and kinesin family member 5B-RET [7,8,9]. The chimeric fusion of RET proteins leads to RET activation by mechanisms described in a previous study [1]. Other forms of oncogenic *RET* alterations are point mutations such as M918T or A883F, resulting in RET activation in the absence of ligand stimulation [6]. Chromosomal rearrangement of *RET* is usually observed in PTC, and point mutations are typically found in MTC [6].

Based on these observations, RET has emerged as an attractive target for thyroid cancer treatments, and thus, RET inhibitors have been actively investigated. Several RET inhibitors have been approved for thyroid cancer therapy by the Food and Drug Administration (FDA). Vandetanib, cabozantinib, lenvatinib, and sorafenib are multikinase inhibitors with RET-inhibitory activity that have been repurposed for thyroid cancer treatments [1]. Recently, the selective RET inhibitors, selpercatinib and pralsetinib (BLU-667), were approved for several indications, including RET-mutant MTC. RXDX-105 and TPX-0046 are currently under investigation [10,11].

Compounds containing an indirubin core skeleton have been reported to inhibit kinases. Various kinases are affected by indirubin derivatives, depending on the chemical modification of the indirubin core skeleton. Kinases inhibited by these derivatives include glycogen synthase kinase 3β, cyclin-dependent kinase, dual-specificity tyrosine phosphorylation-regulated kinase, and Aurora kinases [12,13,14,15]. Our group also previously reported indirubin 3′-oxime derivatives as potent CDK2 and FMS-like tyrosine kinase 3 (FLT3) inhibitors [16,17]. These characteristics of indirubin-3′-oxime derivatives and their function as kinase inhibitors led us to evaluate their RET-inhibitory function. This study describes the characterization of the indirubin-3′-oxime compound LDD-2633 as a potent and novel RET inhibitor with in vitro and in vivo anti-tumor functions.

## 2. Results

### 2.1. LDD-2633 Inhibits the Kinase Activity of RET In Vitro

**Figure 1:**
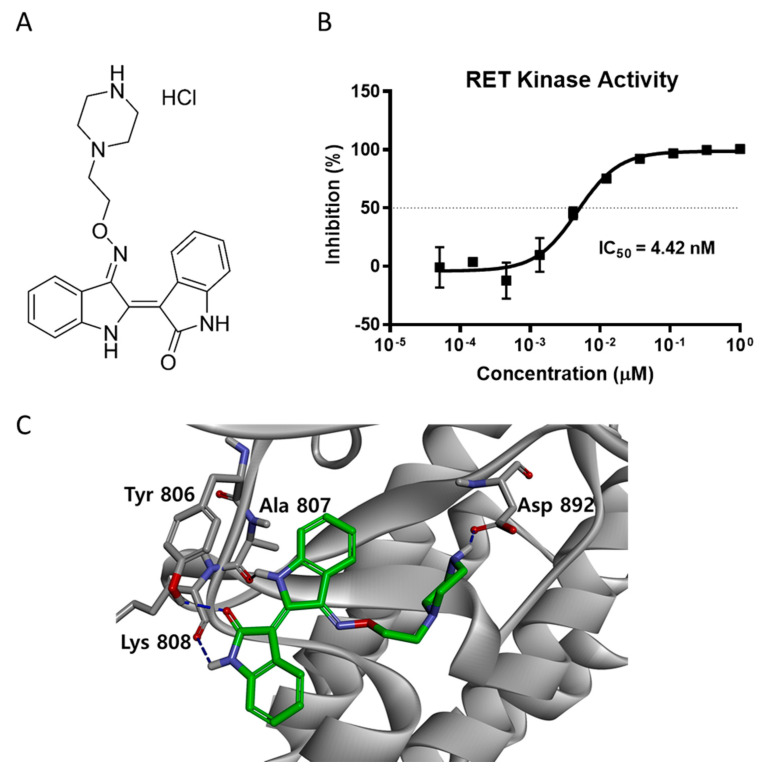
RET kinase activity inhibition by LDD-2633 (**A**) Chemical structure of LDD-2633 (**B**) Effect of LDD-2633 on in vitro RET kinase activity. The TR-FRET method was employed to measure in vitro kinase activity using recombinant RET protein. Data are presented as the mean ± SEM of three independent experiments. (**C**) Binding mode of LDD-2633 in the ATP binding site of RET kinase. Molecular docking was performed using the X-ray crystal structure of the RET protein (PDB code: 2X2K). Residues incorporated by hydrogen bonding in the ATP binding site are denoted in gray color. The network of hydrogen bonds is represented with the blue dashed line.

The indirubin derivative LDD-2633 (Figure 1A) was subjected to an in vitro RET kinase activity assay using RET recombinant protein. The inhibitory activity against RET kinase was measured as described in the Materials and Methods section. LDD-2633 exhibited potent activity against RET kinase with an IC_50_ of 4.42 ± 0.0899 nM (Figure 1B).

To better understand the binding mechanism of LDD-2633, we performed a docking study using the X-ray crystal structure of the phosphorylated RET tyrosine kinase domain (PDB code: 2X2K) [18,19]. LDD-2633 was docked using the CDOCKER protocol in the Discovery Studio 3.5 program. The docking results showed that LDD-2633 successfully docked into the ATP-binding site. The most stable binding mode among the top 10 LDD-2633 docking positions is shown in Figure 1C. The indirubin core skeleton formed two signature hydrogen bonds with the backbone carbonyl groups of the Ala807 and Lys808 residues. Two additional hydrogen bonds were formed between the carbonyl moiety of the indirubin skeleton and the phenolic moiety of the Tyr806 residue in the hinge region. The ethylpiperazine moiety occupied the inner pocket generated by the Asp-Phe-Gly (DFG) motif shift and showed strong hydrogen bonding with the Asp892 residue located in the DFG motif.

From these results, we predicted that the direct inhibition of RET activity by LDD-2633 is mediated by interaction with the ATP-binding site of the RET kinase.

### 2.2. LDD-2633 Suppresses the Growth of TT MTC Cells

**Figure 2:**
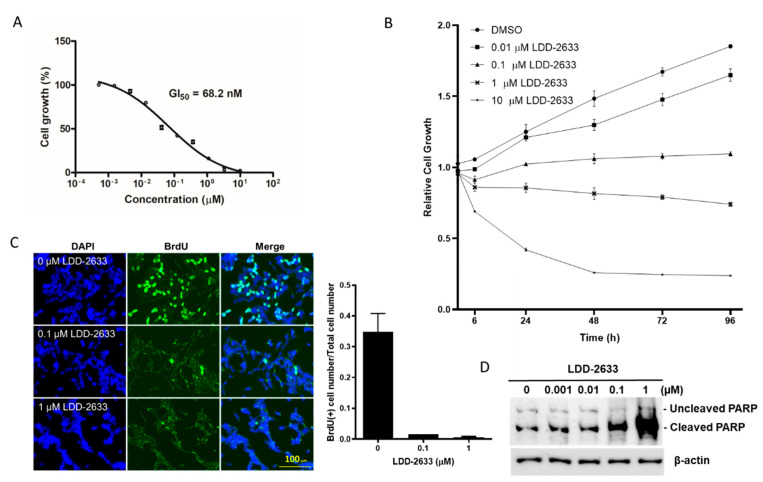
Effect of LDD-2633 on the TT cell growth and apoptosis. (**A**) Cells were treated with LDD-2633 at the indicated concentration for 72 h, and GI_50_ was measured using the EZ-Cytox Cell Viability Assay kit. The data are the mean ± SEM of three independent experiments. (**B**) The cell growth curve was determined in the presence of LDD-2633 at the indicated concentration for up to 96 h using EZ-Cytox Cell Viability Assay kit. The data are the mean ± SEM of three independent experiments. (**C**) Cell proliferation was measured using BrdU incorporation assay. Cells were treated with LDD-2633 at the indicated concentration for 24 h and BrdU incorporation was visualized using BrdU immunofluorescence and DAPI staining. The BrdU-containing cells and total cells stained with DAPI were counted and the ratio was used in the graph. (**D**) Cells were treated with LDD-2633 at the indicated concentrations for 48 h and were then subjected to Western blotting analysis using an antibody to detect both cleaved and uncleaved poly (ADP-ribose) polymerase. Immunoblotting analysis of β-actin was presented as the loading control.

TT cells are a cancer cell line that originates from the thyroid medulla and harbor a cysteine 634 to tryptophan (C634W) mutation of *RET*, which is a constitutively activating point mutation [20]. The treatment of TT cells with LDD-2633 resulted in potent growth inhibition with a GI_50_ of 68.2 ± 2.92 nM (Figure 2A). Cell growth curves were obtained for up to 96 h in the presence of LDD-2633 at 0.01, 0.1, 1, and 10 µM concentration along with dimethyl sulfoxide (DMSO) control (Figure 2B). Relative cell numbers in the absence of LDD-2633 increased 1.9 fold at 96 h, while the addition of LDD-2633 dose-dependently suppressed cell growth. Cell numbers almost did not change at 0.1 µM LDD-2633, but was reduced after 1 µM and 10 µM LDD-2633 treatment.

The effects of LDD-2633 on cell proliferation were determined using BrdU incorporation assay (Figure 2C). Cells in the S phase were labeled with BrdU and incorporated BrdU was visualized using immunofluorescence. BrdU- and DAPI-stained cells were counted and the ratio was calculated. Treatment with LDD-2633 strongly reduced the number of BrdU-positive cells, indicating decreased cell proliferation by LDD-2633. A reduction of 95.2% and 98.4% in BrdU-positive cell population was observed by 0.1 µM and 1 µM LDD-2633, respectively.

The effect of LDD-2633 on apoptosis was also evaluated by measuring the expression of cleaved poly (ADP-ribose) polymerase (PARP). LDD-2633 was found to induce PARP cleavage in a dose-dependent manner, as shown in Figure 2D. At 1 µM, LDD-2633 cleaved considerable amount of PARP, indicating a major contribution of apoptotic cell death to TT cell growth suppression. Taken together, Figure 1 results indicate that LDD-2633 inhibited TT cell growth by suppressing proliferation and inducing apoptosis.

### 2.3. LDD-2633 Suppresses RET Signaling Pathway

**Figure 3:**
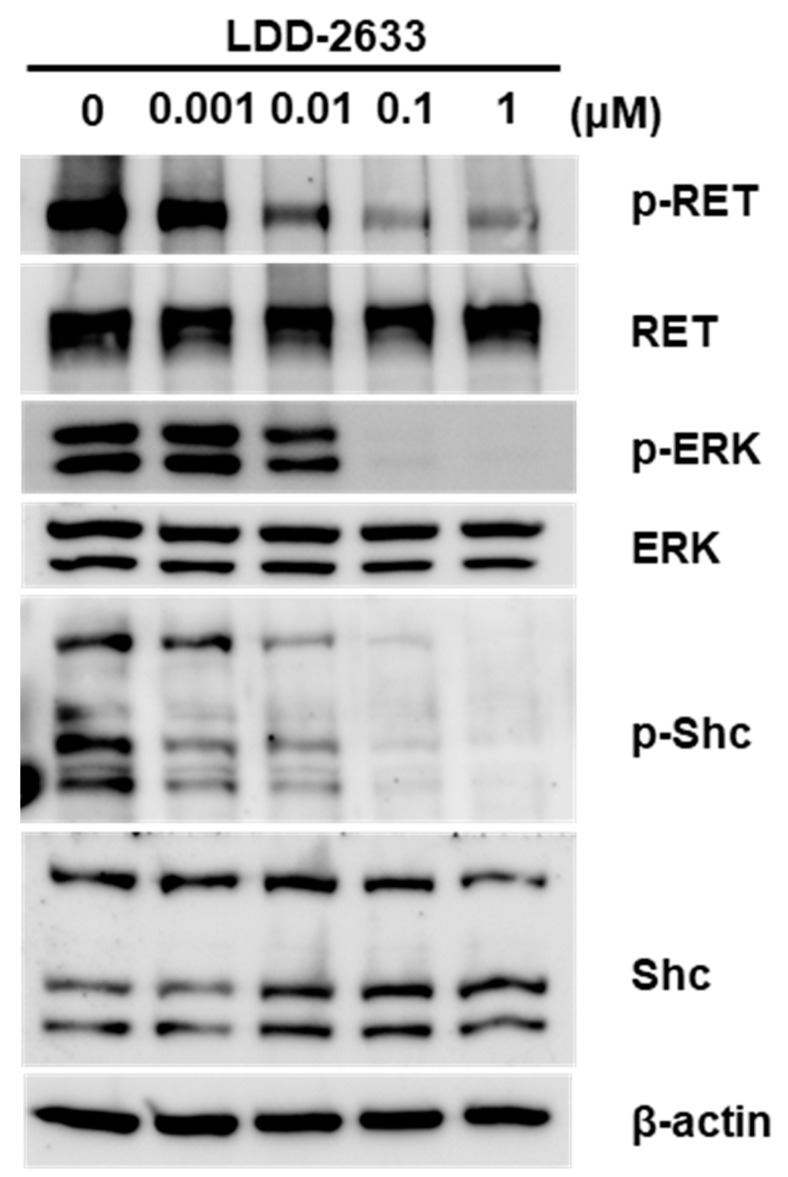
Effect of LDD-2633 on the RET signaling pathway. TT cells were treated with LDD-2633 at the indicated concentration for 48 h. Western blotting experiments were then performed using the indicated antibodies. Blotting images are representative of three independent experiments. Immunoblotting analysis of β-actin was presented as the loading control.

To examine the effects of LDD-2633 on the RET signaling pathway in TT cells, Western blotting analyses for RET and the downstream effectors were performed. The phosphorylation level of RET kinase was measured using an antibody specific to the phosphorylated RET (p-RET) protein. Dose-dependent downregulation of p-RET was observed upon treatment with LDD-2633 (Figure 3). The phosphorylation of Shc and ERK1/2, which are downstream molecules of RET, also decreased upon treatment with LDD-2633 (Figure 3). These results confirmed the inhibition of RET activity by LDD-2633.

### 2.4. LDD-2633 Exerts Anti-Tumor Effects In Vivo in TT Cell Xenografts

**Figure 4:**
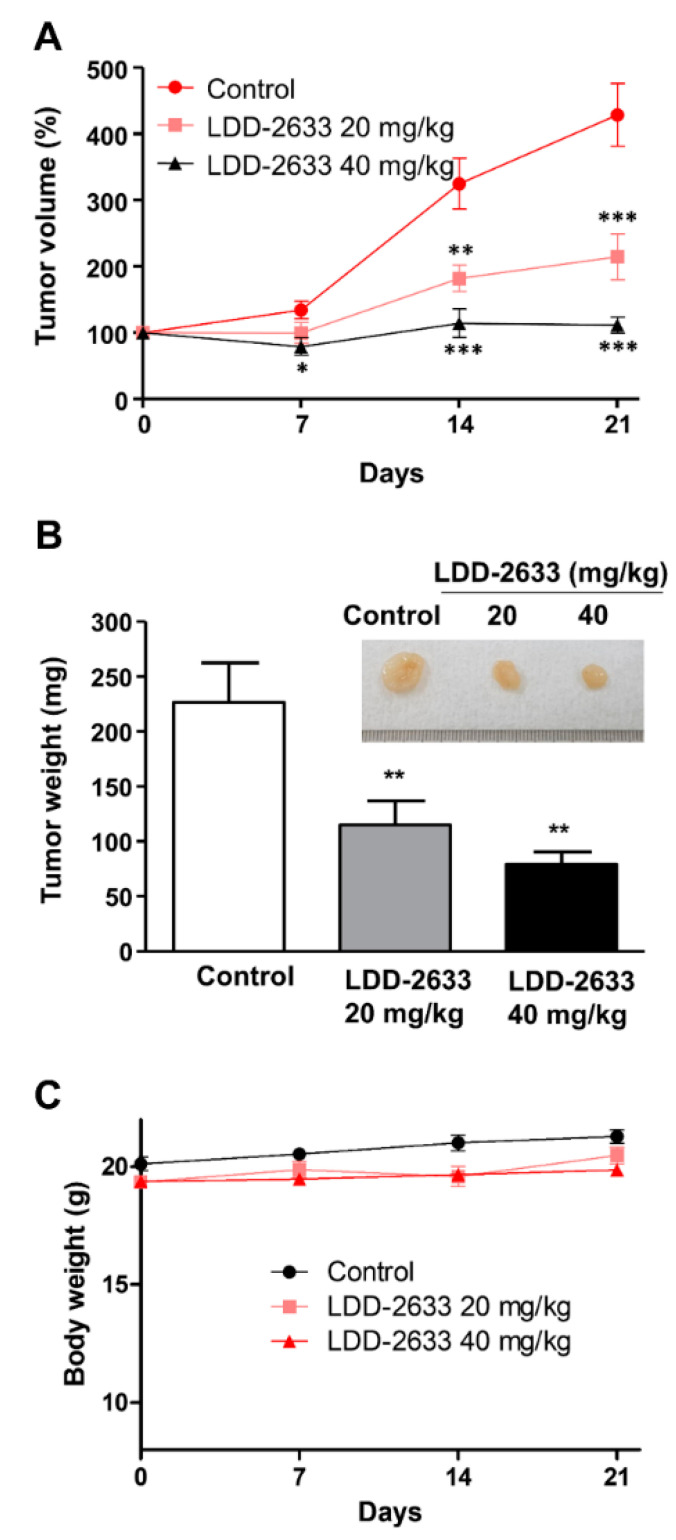
In vivo anti-tumor efficacy of LDD-2633. Tumor xenografts were generated by inoculating TT cells into BALB/c nu/nu mice, and the tumors were allowed to grow to the size of 100 mm^3^ (*n* = 8). LDD-2633 (20 or 40 mg/kg) or PBS control was administered to the mice by oral gavage for 21 days. (**A**) The tumor size was measured for the indicated time after starting drug administration. Tumor volume was calculated using the following formula: V (volume) = X (length) × D (width)^2^/2. (**B**) Tumor xenografts were isolated and tumor weights were measured on day 21. Representative photographic images of tumors are shown in the inset. (**C**) Mice body weights were measured at the indicated time. Results represent the means ± standard errors of mean. The symbols *, **, and *** indicate *p* < 0.05, *p* < 0.01, and *p* < 0.001, respectively.

The in vivo anti-tumor effects of LDD-2633 were examined using tumor xenografts, generated in athymic mice via TT cell inoculation. LDD-2633 and PBS control were administered by oral gavage. Tumor size was measured for 21 days after starting drug treatment. As shown in Figure 4, LDD-2633 suppressed tumor growth in a dose-dependent manner. At a dose of 40 mg/kg of LDD-2633, tumor volume did not increase during the drug treatment period. Tumor weights recorded on day 21 also indicated that LDD-2633 induced tumor growth suppression. The results showed that the average tumor weights in the 20 mg/kg LDD-2633 and 40 mg/kg LDD-2633 groups were 50.1% and 34.8%, respectively, of those in the control group (Figure 4B). The body weight of each mouse was measured to determine the toxicity of LDD-2633 and was found to remain unchanged over the course of treatments (Figure 4C). These in vivo anti-tumor effects indicated the potential of LDD-2633 as a candidate for the development of thyroid cancer therapy.

## 3. Discussion

Since the discovery of the *RET* gene aberration, there have been studies on the development of RET kinase inhibitors for treating thyroid cancer. Currently, drugs such as vandetanib, cabozantinib, sorafenib, lenvatinib, selpercatinib, and pralsetinib are FDA-approved RET kinase inhibitors for the treatment of thyroid cancers. These drugs, except selpercatinib, are multikinase inhibitors originally developed as anti-angiogenesis agents. The RET-inhibitory activity of these drugs was discovered later, driving their development as anti-thyroid cancer agents. At the time of vandetanib approval (i.e., 2011), no pharmacological therapeutic options were available for MTC; the only available treatments were radiation therapy and surgery. With the introduction of vandetanib, and subsequently lenvatinib, MTC pharmacotherapy became possible for unresectable MTC cases.

Vandetanib, cabozantinib, and sorafenib have inhibitory activity against FLT3 kinase along with growth inhibitory activity against acute myeloid leukemia cells with FLT3 mutation [21,22,23]. These drugs primarily inhibit vascular endothelial growth factor receptor 2 to induce anti-angiogenic effects. The activity of multiple kinases, including FLT3, is also affected, and this multikinase inhibition might contribute to the anti-tumor effects of vandetanib, cabozantinib, and sorafenib. Similar to other multikinase inhibitors, LDD-2633 is a potent inhibitor of both FLT3 and RET kinases [24]. LDD-1937, a potent FLT3 inhibitor we previously reported [17], also strongly inhibits RET activity with an IC_50_ of 3.80 nM (unpublished data). The ATP-binding sites of FLT3 and RET kinases for drug interaction are presumed to be similar, given the similar kinase inhibition profiles of multikinase inhibitors.

The selective RET inhibitor selpercatinib was approved by the FDA in May 2020 while this manuscript was being drafted. Selpercatinib is indicated in *RET* fusion-positive advanced non-small lung cancer (NSCLC), *RET*-mutant MTC, and *RET* fusion-positive thyroid cancer. Pralsetinib, another selective RET inhibitor, was also approved in September 2020, for *RET* fusion-positive NSCLC [25]. Indications of pralsetinib for *RET* mutant MTC and *RET* fusion-positive thyroid cancer are under review. The robust response produced by selpercatinib and pralsetinib will benefit patients with *RET*-mutant cancers.

As shown by selpercatinib and pralsetinib, another important indication for RET inhibitors is NSCLC with *RET* fusion. Chromosomal rearrangements resulting in RET fusion have been identified in 1% to 2% of NSCLCs [2]. Selpercatinib has been indicated in *RET* fusion-positive NSCLC as well as thyroid cancers. A novel drug development approach called tissue-agnostic therapy has allowed fast approval of these drugs by assessing relatively small number of patients in clinical studies. Three different types of tumors (NSCLC, MTC, and PTC), with the common biomarker of RET aberration, were tested together. The tissue-agnostic approach, which is based on biomarkers and is independent of tumor types, has been employed for the development of selpercatinib and pralsetinib [26]. The anti-tumor effects of LDD-2633 may be extended to NSCLCs harboring RET fusion proteins.

Growth suppression by LDD-2633 (Figure 2A) can be mediated via several mechanisms: suppression of proliferation, apoptosis, and senescence are well-known mechanisms in tumor growth suppression. As indicated in Figure 2C, BrdU-positive cells indicating cell population at S phase dramatically reduced by LDD-2633 treatment. Apoptotic cells increased as shown by high levels of PARP cleavage (Figure 2D). However, cellular senescence was not detected following LDD-2633 treatment (data not shown).

The in vivo anti-tumor activity of LDD-2633 in TT cell xenografts was also measured (Figure 4). The oral bioavailability of LDD-2633 was 42.6% [24], thus LDD-2633 was orally administered in xenograft study. Tumor growth was suppressed; however, no tumor remission was not observed, as can be seen from the change in tumor size over the course of LDD-2633 treatments (Figure 4A). It was not clear if the 40 mg/kg dose of LDD-2633 was insufficient for complete remission. Therefore, dose escalation of LDD-2633 is required to determine if tumor remission can be induced by this drug.

In this study, the indirubin derivative LDD-2633 was characterized as a novel RET inhibitor with anticancer activity against MTC. LDD-2633 exhibited RET kinase inhibitory activity in vitro in the MTC cell line TT. Moreover, TT cell growth was suppressed by treatment with LDD-2633, which was consistent with the observed inhibition of RET activity. LDD-2633 also exhibited anti-tumor effects in TT cell xenografts in mice. These results demonstrate the potential of LDD-2633 as an anticancer agent for medullary thyroid cancer.

## 4. Materials and Methods

### 4.1. Compounds

LDD-2633, (2Z,3E)-3-((2-(piperazin-1-yl)ethoxy)imino)-[2,3′-biindolinylidene]-2′-one hydrochloride, was designed and synthesized. The compounds were dissolved in DMSO at a concentration of 10 mmol/L and stored at −20 °C. Their synthetic schemes and structure-activity relationships have been outlined in a separate study [24].

### 4.2. Cell Culture

The human TT thyroid medullary carcinoma cell line was purchased from the American Type Culture Collection (Manassas, VA, USA). TT cells were grown as monolayers in RPMI-1640 medium (Sigma Co., St. Louis, MO, USA) supplemented with 15% fetal bovine serum and 1% penicillin/streptomycin (Hyclone, Logan, UT, USA) and maintained at 37 °C in a humidified atmosphere with 5% CO_2_.

### 4.3. Cell Viability Assay and BrdU Incorporation Assay

Cell viability was assessed by tetrazolium-based assay using the EZ-Cytox Cell Viability Assay kit (DaeilLab, Seoul, Korea). Cells were seeded in 96-well plates (5000 cells/well) and incubated with either the compounds or DMSO as a negative control. After 72 h of incubation, cell viability was measured by adding 15 μL of EZ-Cytox reagent to each well. After incubating for 4 h, absorbance at 450 nm was measured using a Victor multi-label reader (Perkin Elmer, Waltham, MA, USA). GI₅₀ was calculated by nonlinear regression using Prism version 8.4.3 (GraphPad, La Jolla, CA, USA).

To assess cell growth curve, TT cells (5000 cells/well) were seeded in 24-well plates and incubated with either LDD-2633 compound or DMSO. After 6 h, 24 h, 48 h, and 96 h of incubation, cells were detached and counted using hemocytometer.

For 5-bromo-2′-deoxyuridine (BrdU) incorporation assay, 1 × 10^6^ cells in 6-well plates were incubated with 10 μM BrdU (HY-15910; MedChemExpress, Monmouth, NJ, USA) for 18 h. Cells were washed and fixed with 2% formaldehyde for 15 min, permeabilized with 0.2% Triton X-100 solution for 5 min and then treated with 2 M HCl for 30 min. After blocking with 10% FBS in phosphate-buffered saline (PBS) for 30 min, the cells were incubated with anti-BrdU specific primary antibody (Santa Cruz, sc-32323; 1:50) in immunofluorescence buffer (IF buffer; 0.75% glycine in PBS). The cells were kept at room temperature for 2 h and then washed three times with IF buffer. The primary antigen-antibody reaction was detected with Alexa flour-488 conjugated secondary antibody (Thermo Fisher Scientific, Waltham, MA, USA, #A-11001; 1:500) in IF buffer. The cells were incubated with the secondary antibody together with 5 μg/mL DAPI counterstain at 25 °C for 1 h, washed and observed using Axiovert 200 fluorescence microscope (Carl Zeiss, Jena, Germany).

### 4.4. Molecular Modeling

Molecular docking was performed using CDOCKER, a CHARMM-based molecular dynamics docking algorithm in Discovery Studio 3.5 (BIOVIA Corp, San Diego, CA, USA). The X-ray crystal structure of the RET kinase was obtained from the Protein Data Bank (PDB code: 2X2K). The ATP-binding site was chosen as the active site, and the radius of this site was set to 12 Å. Docking was performed using all default parameters. The calculated CDOCKER interaction energy differences were less than 10%.

### 4.5. In Vitro Kinase Assay

Time-resolved fluorescence-based HTRF KinEASE-TK (Cisbio, Codolet, France) was used to evaluate the RET kinase activity as previously described [27]. The reaction was performed in a 96-well plate with a kinase reaction mixture containing 0.1 μM TK-substrate biotin, 100 μM ATP, 1 ng of RET kinase (Millipore, Darmstadt, Germany), and a serial three-fold dilution of the test compound in a kinase reaction buffer (50 mM HEPES; pH 7.0, 5 mM MgCl_2_, 1 mM DTT, 0.1 mM orthovanadate, 0.01% bovine serum albumin [BSA], and 0.02% NaN_3_). After the addition of the detection reagents, time-resolved fluorescence energy transfer signals were detected using a Victor multi-label reader (Perkin Elmer) at 615 nm and 655 nm. The dose-response curve was fitted by nonlinear regression, and the IC₅₀ was computed using Prism version 8.4.3.

### 4.6. Immunoblotting Analysis

Cells were lysed in sodium dodecyl sulfate (SDS) lysis buffer (200 mM Tris-HCl [pH 7.4], 10% glycerol and 0.4% SDS), and protein concentrations were measured using the SMART bicinchoninic acid protein assay kit (iNtRON Biotechnology, Seongnam, Korea). Equal amounts of proteins were resolved using SDS-polyacrylamide gel electrophoresis (8–15% acrylamide) and transferred to polyvinylidene difluoride membranes (Merck Millipore Ltd., Tullagreen, Carrigtwohill, Germany). Next, the membrane was blocked by incubating the membranes in 1× Tris-Buffered Saline, 0.1% Tween^®^ 20 Detergent (TBST), 5% nonfat dry milk (NDM) or 5% bovine serum albumin (BSA) for 30 min at 22 °C to prevent nonspecific binding of antibodies to the surface of membrane. The membranes were then incubated overnight at 4 °C in blocking buffer with one of following primary antibodies; blocking agents are indicated for individual antibodies: antibodies against p-RET (Tyr905, #3221; 1:1000; BSA), RET (#3223; 1:1000; NDM), phosphorylated-p44/42 MAPK (1:1000, ERK1/2; #4370; BSA), phosphorylated Shc (Tyr 239/240, #2434; 1:1000; BSA), and PARP (#9542; 1:1000; NDM) were purchased from Cell Signaling Technology (Danvers, MA, USA); anti-ERK1/2 (sc-135900, 1:1000; NDM) was purchased from Santa Cruz Biotechnology (Santa Cruz, CA, USA); anti-Shc (#610878; 1:1000; NDM) was purchased from BD Biosciences (Franklin Lakes, NJ, USA); an antibody against β-actin (Sigma-Aldrich, St. Louis, MO, USA, #5441; 1:5000; NDM) was used as a loading control. On the following day, the membranes were washed with 1× TBST buffer and incubated in blocking buffer with goat anti-rabbit IgG (Jackson Laboratory; Bar Harbor, ME, USA, 111-035-003; 1:5000) or goat anti-mouse IgG (Jackson Laboratory, 115-035-003; 1:5000) secondary antibodies for 1 h at 22 °C. Subsequently, the membranes were washed with 1× TBST buffer, and immunoblot signals were detected using the ECL Select Western Blotting Detection Reagent (Amersham ECL Select, GE Healthcare, London, UK).

### 4.7. Mouse Tumor Xenograft

To establish thyroid tumor mice, 5 × 10^6^ TT cells were suspended in 200 μL of PBS and inoculated subcutaneously in the flanks of seven-weeks-old female BALB/c *nu*/*nu* (athymic nude) mice. When the tumor reached a mean volume of 100 mm^3^ (approximately 14 days after inoculation), the mice were randomly divided into three groups (*n* = 8 per group) and were administered either 20 or 40 mg/kg of LDD-2633 in PBS (pH 7.4) or pure PBS (control) by oral gavage. The drug or control PBS was administered daily for 21 days. Tumor size was measured twice a week for 21 days, and tumor volumes were calculated using the following formula:

$$V=\frac{XD^{2}}{2}$$

where *V* is the volume (mm^3^), *X* is the length (mm), and *D* is the width of the tumor (mm). After 21 days, the mice were sacrificed, and tumor weights (mg) were measured.

## Data Availability

Data sharing is not applicable to this article.

## References

[1] Tuttle R.M., Ball D.W., Byrd D., Dilawari R.A., Doherty G.M., Duh Q.Y., Ehya H., Farrar W.B., Haddad R.I., Kandeel F. (2010). Wirth, and Network National Comprehensive Cancer—“Thyroid Carcinoma”. J. Natl. Compr. Cancer Netw..

[2] Drilon A., Hu Z.I., Lai G.G.Y., Tan D.S.W. (2017). Targeting RET-driven cancers: Lessons from evolving preclinical and clinical landscapes. Nat. Rev. Clin. Oncol..

[3] DeVita V.T., Lawrence T.S., Rosenberg S.A. (2015). Devita, Hellman, and Rosenberg’s Cancer: Principles & Practice of Oncology.

[4] Durante C., Haddy N., Baudin E., Leboulleux S., Hartl D., Travagli J.P., Caillou B., Ricard M., Lumbroso J.D., De Vathaire F. (2006). Long-Term Outcome of 444 Patients with Distant Metastases from Papillary and Follicular Thyroid Carcinoma: Benefits and Limits of Radioiodine Therapy. J. Clin. Endocrinol. Metab..

[5] Roman S., Lin R., Sosa J.A. (2006). Prognosis of Medullary Thyroid Carcinoma: Demographic, Clinical, and Pathologic Predictors of Survival in 1252 Cases. Cancer.

[6] Romei C., Ciampi R., Elisei C.R.R.C.R. (2016). A comprehensive overview of the role of the RET proto-oncogene in thyroid carcinoma. Nat. Rev. Endocrinol..

[7] Kohno T., Ichikawa H., Totoki Y., Yasuda K., Hiramoto M., Nammo T., Sakamoto H., Tsuta K., Furuta K., Shimada Y. (2012). KIF5B-RET fusions in lung adenocarcinoma. Nat. Med..

[8] Nikiforov Y.E., Rowland J.M., Bove K.E., Monforte-Munoz H., Fagin J.A. (1997). Distinct pattern of ret oncogene rearrangements in morphological variants of radiation-induced and sporadic thyroid papillary carcinomas in children. Cancer Res..

[9] Nikiforov Y.E. (2002). RET/PTC Rearrangement in Thyroid Tumors. Endocr. Pathol..

[10] Li G.G., Somwar R., Joseph J., Smith R.S., Hayashi T., Martin L., Franovic A., Schairer A., Martin E., Riely G.J. (2017). Antitumor Activity of RXDX-105 in Multiple Cancer Types with RET Rearrangements or Mutations. Clin. Cancer Res..

[11] Subbiah V., Cote G.J. (2020). Advances in Targeting RET-Dependent Cancers. Cancer Discov..

[12] Leclerc S., Garnier M., Hoessel R., Marko D., Bibb J.A., Snyder G.L., Greengard P., Biernat J., Wu Y.Z., Mandelkow E.M. (2001). Indirubins Inhibit Glycogen Synthase Kinase-3 Beta and Cdk5/P25, Two Protein Kinases Involved in Abnormal Tau Phosphorylation in Alzheimer’s Disease. A Property Common to Most Cyclin-Dependent Kinase Inhibitors?. J. Biol. Chem..

[13] Hoessel R., Leclerc S., Endicott J.A., Nobel M.E.M., Lawrie A., Tunnah P., Leost M., Damiens E., Marie D., Marko D. (1999). Indirubin, the active constituent of a Chinese antileukaemia medicine, inhibits cyclin-dependent kinases. Nat. Cell Biol..

[14] Myrianthopoulos V., Kritsanida M., Gaboriaud-Kolar N., Magiatis P., Ferandin Y., Durieu E., Lozach O., Cappel D., Soundararajan M., Filippakopoulos P. (2012). Novel Inverse Binding Mode of Indirubin Derivatives Yields Improved Selectivity for DYRK Kinases. ACS Med. Chem. Lett..

[15] Ndolo K.M., Park K.R., Lee H.J., Bin Yoon K., Kim Y.-C., Han S.-Y. (2017). Characterization of the Indirubin Derivative LDD970 as a Small Molecule Aurora Kinase A Inhibitor in Human Colorectal Cancer Cells. Immune Netw..

[16] Choi S.-J., Lee J.E., Jeong S.-Y., Im I., Lee S.-D., Lee E.-J., Lee S.K., Kwon S.-M., Ahn S.-G., Yoon J.-H. (2010). 5,5′-Substituted Indirubin-3′-oxime Derivatives as Potent Cyclin-Dependent Kinase Inhibitors with Anticancer Activity. J. Med. Chem..

[17] Lee H.J., Lee J., Jeong P., Choi J., Baek J., Ahn S.J., Moon Y., Heo J.D., Choi Y.H., Chin Y.W. (2018). Discovery of a Flt3 Inhibitor Ldd1937 as an Anti-Leukemic Agent for Acute Myeloid Leukemia. Oncotarget.

[18] Mologni L., Rostagno R., Brussolo S., Knowles P.P., Kjaer S., Murray-Rust J., Rosso E., Zambon A., Scapozza L., McDonald N.Q. (2010). Synthesis, structure–activity relationship and crystallographic studies of 3-substituted indolin-2-one RET inhibitors. Bioorganic Med. Chem..

[19] Knowles P.P., Murray-Rust J., Kjaer S., Scott R.P., Hanrahan S., Santoro M., Ibáñez C.F., McDonald N.Q. (2006). Structure and Chemical Inhibition of the RET Tyrosine Kinase Domain. J. Biol. Chem..

[20] Carlomagno F., Salvatore D., Santoro M., De Franciscis V., Quadro L., Panariello L., Colantuoni V., Fusco A. (1995). Point mutation of the RET proto-oncogene in the TT human medullary thyroid carcinoma cell line. Biochem. Biophys. Res. Commun..

[21] Nishioka C., Ikezoe T., Takeshita A., Yang J., Tasaka T., Yang Y., Kuwayama Y., Komatsu N., Togitani K., Koeffler H.P. (2007). ZD6474 induces growth arrest and apoptosis of human leukemia cells, which is enhanced by concomitant use of a novel MEK inhibitor, AZD6244. Leukemia.

[22] Lu J.-W., Wang A.-N., Liao H.-A., Chen C.-Y., Hou H.-A., Hu C.-Y., Tien H.-F., Ou D.-L., Lin L.-I. (2016). Cabozantinib is selectively cytotoxic in acute myeloid leukemia cells with FLT3-internal tandem duplication (FLT3-ITD). Cancer Lett..

[23] Liu T., Ivaturi V., Sabato P., Gobburu J.V.S., Greer J.M., Wright J.J., Smith B.D., Pratz K.W., Rudek M.A., on behalf of the ETCTN-6745 study team (2018). Sorafenib Dose Recommendation in Acute Myeloid Leukemia Based on Exposure-FLT3 Relationship. Clin. Transl. Sci..

[24] Jeong P., Moon Y., Lee J.-H., Lee S.-D., Park J., Lee J., Kim J., Lee H.J., Kim N.Y., Choi J. (2020). Discovery of orally active indirubin-3′-oxime derivatives as potent type 1 FLT3 inhibitors for acute myeloid leukemia. Eur. J. Med. Chem..

[25] Markham A. (2020). Pralsetinib: First Approval. Drugs.

[26] Looney A.-M., Nawaz K., Webster R.M. (2020). Tumour-agnostic therapies. Nat. Rev. Drug Discov..

[27] Ndolo K.M., An S.J., Park K.R., Lee H.J., Bin Yoon K., Kim Y.-C., Han S.-Y. (2019). Discovery of an Indirubin Derivative as a Novel c-Met Kinase Inhibitor with In Vitro Anti-Tumor Effects. Biomol. Ther..

